# BCG as a Case Study for Precision Vaccine Development: Lessons From Vaccine Heterogeneity, Trained Immunity, and Immune Ontogeny

**DOI:** 10.3389/fmicb.2020.00332

**Published:** 2020-03-11

**Authors:** Asimenia Angelidou, Joann Diray-Arce, Maria Giulia Conti, Kinga K. Smolen, Simon Daniël van Haren, David J. Dowling, Robert N. Husson, Ofer Levy

**Affiliations:** ^1^Division of Newborn Medicine, Boston Children’s Hospital and Beth Israel Deaconess Medical Center, Boston, MA, United States; ^2^Precision Vaccines Program, Boston Children’s Hospital, Boston, MA, United States; ^3^Harvard Medical School, Boston, MA, United States; ^4^Division of Infectious Diseases, Boston Children’s Hospital, Boston, MA, United States; ^5^Department of Maternal and Child Health, Sapienza University of Rome, Rome, Italy

**Keywords:** BCG formulation, immunogenicity, mycobacteria, ontogeny, trained immunity, vaccine

## Abstract

Vaccines have been traditionally developed with the presumption that they exert identical immunogenicity regardless of target population and that they provide protection solely against their target pathogen. However, it is increasingly appreciated that vaccines can have off-target effects and that vaccine immunogenicity can vary substantially with demographic factors such as age and sex. Bacille Calmette-Guérin (BCG), the live attenuated *Mycobacterium bovis* vaccine against tuberculosis (TB), represents a key example of these concepts. BCG vaccines are manufactured under different conditions across the globe generating divergent formulations. Epidemiologic studies have linked early life immunization with certain BCG formulations to an unanticipated reduction (∼50%) in all-cause mortality, especially in low birthweight males, greatly exceeding that attributable to TB prevention. This mortality benefit has been related to prevention of sepsis and respiratory infections suggesting that BCG induces “heterologous” protection against unrelated pathogens. Proposed mechanisms for heterologous protection include vaccine-induced immunometabolic shifts, epigenetic reprogramming of innate cell populations, and modulation of hematopoietic stem cell progenitors resulting in altered responses to subsequent stimuli, a phenomenon termed “trained immunity.” In addition to genetic differences, licensed BCG formulations differ markedly in content of viable mycobacteria key for innate immune activation, potentially contributing to differences in the ability of these diverse formulations to induce TB-specific and heterologous protection. BCG immunomodulatory properties have also sparked interest in its potential use to prevent or alleviate autoimmune and inflammatory diseases, including type 1 diabetes mellitus and multiple sclerosis. BCG can also serve as a model: nanoparticle vaccine formulations incorporating Toll-like receptor 8 agonists can mimic some of BCG’s innate immune activation, suggesting that aspects of BCG’s effects can be induced with non-replicating stimuli. Overall, BCG represents a paradigm for precision vaccinology, lessons from which will help inform next generation vaccines.

## The BCG Vaccine

BCG, the live attenuated vaccine against tuberculosis (TB), is one of the world’s most widely used vaccines (Andersen and Doherty, 2005; Aaby et al., 2010) and continues to be the only vaccine used to prevent TB. It contains an attenuated strain of the bovine tubercle bacillus *Mycobacterium bovis* and was first introduced in humans in 1921. BCG is used to induce immunity against TB and is part of the World Health Organization’s (WHO’s) Expanded Program on Immunization (EPI) with more than 100 million children vaccinated with BCG every year (World Health and Organization, 2004). Universal vaccination at birth with a single dose of BCG is recommended in developing countries where TB is highly endemic or where there is high risk of exposure to TB. Because of the declining incidence of TB in Europe and the United States, BCG immunization is mostly recommended for high-risk groups in these regions. A database of global BCG vaccination policies and practices can be found online^1^ (Zwerling et al., 2011).

BCG has an excellent and long-standing record of safety (Saroha et al., 2015) and tolerability with the most common adverse effect being regional suppurative lymphadenitis, which is a rare occurrence. The most serious complication of BCG vaccination is disseminated BCG infection (rate of 0.06–1.56 cases per million doses of vaccine administered), occurring primarily in immunocompromised individuals, including neonates with undiagnosed primary immunodeficiency (Marciano et al., 2014). Possible factors affecting the rate of adverse reactions include the BCG dose, vaccine strain, and method of vaccine administration (Lotte et al., 1984).

### TB-Specific Protection Conferred by BCG Vaccine

Multiple aspects of BCG remain incompletely characterized, including its overall efficacy, duration of protective immunity, and how age at vaccination affects protection. The variability of BCG protective efficacy has been systematically studied (Mangtani et al., 2014). In children, BCG confers 58% protection against progression of TB infection to disease (Roy et al., 2014) and ∼80% protection against severe or disseminated forms of TB, such as meningitis and miliary disease (Rodrigues et al., 1993; Trunz et al., 2006). Decreasing BCG coverage in European countries was followed by an increased incidence of TB (Romanus et al., 1992; Kelly et al., 1997) and other mycobacterial diseases (Romanus et al., 1995; Dowling et al., 2017). In adults, BCG reduces the risk of pulmonary TB by ∼50% but has variable efficacy in different populations (Colditz et al., 1994; Brewer, 2000). In summary, across many studies BCG efficacy is variable, with some studies showing minimal benefit, while in others it appears to provide limited protection against infection and progression to TB disease. BCG vaccination has no sizeable impact on TB transmission dynamics as its effectiveness has been mainly demonstrated in childhood, when TB is rarely contagious (Loeffler, 2003).

BCG is considered a ‘self-adjuvanted’ vaccine, as components of the formulation capable of engaging multiple Pattern Recognition Receptors (PRRs), including Toll-like receptor (TLR)2 and TLR4 (Heldwein et al., 2003), TLR8 (Dowling et al., 2017), as well as the C-type lectin receptors Dectin-1 and Mincle (Yadav and Schorey, 2006; Matsunaga and Moody, 2009; Schoenen et al., 2010) are thought to enhance vaccine-induced immunity. Unlike hepatitis B vaccine which requires multiple doses to achieve lymphoproliferation, BCG induces single shot lymphoproliferation (Sanchez-Schmitz et al., 2018). Most recently, in an Indian adult human cohort, a hypermorphic gain of function single nucleotide polymorphism in TLR8, a PRR that is activated by microbial single stranded RNA, was associated with improved BCG vaccine-mediated protection against pulmonary TB (Ugolini et al., 2018).

BCG-induced protection against TB is, at least in part, attributed to a T-helper (Th)1 response. BCG elicits a Th1 cell response in adults, and overcomes the Th2 immune bias present in infants, by inducing adult-like IFNγ responses (Marchant et al., 1999). IFNγ production to many stimuli is muted in newborn T cells, however IFNγ can be produced *in vitro* by neonatal NK cells in response to live microbial stimuli such as BCG after priming with recombinant IFNγ, at least for certain geographic populations (van den Biggelaar et al., 2009). In BCG-vaccinated infants, unconventional gamma-delta (γδ) T cells are also increasingly recognized as a source of IFNγ production (Zufferey et al., 2013), in addition to their bridging role between innate and adaptive immunity against TB infection (Meraviglia et al., 2011). Although protective immunity against TB requires IFNγ responses, a direct association between the concentrations of vaccine-induced IFNγ responses and degree of immune protection has not been seen (Hoft et al., 2002). Further, recent evidence suggests that IFNγ-independent immune responses, including generation of highly avid antibodies and CD40L+/CD154+ T cells, are associated with absence of TB disease in highly exposed contacts of persons with highly infectious TB, though the role of these responses in protection is not clear (Lu et al., 2019). After boost vaccination with a candidate TB vaccine, MVA85A, BCG-induced protection against TB was not enhanced in infants despite more durable T cell responses (Tameris et al., 2013). However, weak immunogenicity was also noted in this trial. Furthermore, dysregulated or excessive CD4^+^ T cell activation can enhance host susceptibility to *Mycobacterium tuberculosis* (Mtb) infection; as such, effector T cell responses must be tightly regulated for host survival to TB (Tzelepis et al., 2018). Although anti-BCG T cell-mediated immunity alone is not adequate to confer protection from TB infection and disease, it can serve as an immune correlate of TB infection and disease risk (Kagina et al., 2010; Fletcher et al., 2016). Parameters such as presence and size of BCG scar and delayed-type hypersensitivity do not predict protective efficacy in humans (Ota et al., 2006; Kagina et al., 2010; Fletcher et al., 2016).

*Mycobacterium bovis* BCG infection induces macrophage production of GM-CSF that may contribute to the host response against mycobacterial infection by favoring macrophage M1 polarization (Benmerzoug et al., 2018). GM-CSF and IFNγ may have an additive effect in promoting macrophage control of intracellular bacterial replication (Rothchild et al., 2017). GM-CSF is produced by a variety of cells, including macrophages and parenchyma cells. It stimulates differentiation of myeloid progenitors into macrophages and neutrophils, regulates hematopoietic cell proliferation and differentiation, and modulates the function of mature hematopoietic cells (Martinez and Gordon, 2014). Clinical observations linking the presence of anti-GM-CSF autoantibodies with susceptibility to cryptococcal meningitis and pulmonary TB support an important role for GM-CSF for host defense against infection (Rosen et al., 2013), and Mtb infection in particular. In addition to activating macrophages to limit the intracellular growth of Mtb *in vitro* (Denis and Ghadirian, 1990), proposed antimicrobial mechanisms of GM-CSF include preserving the integrity of alveolar epithelial cells, regulating cellular lipid metabolism in alveolar macrophages (Rothchild et al., 2017) and facilitating containment of virulent mycobacteria in pulmonary granulomas (Szeliga et al., 2008). Interestingly, GM-CSF along with IL-3 priming of CD14+ human monocytes enhanced TNF production and monocyte renewal (as evaluated by the degree of cell confluency and increased cell number by fluorescence and time-lapse microscopy) upon subsequent LPS stimulation, indicating a potential mechanism of trained immunity (Borriello et al., 2016). As detailed in the following sections, trained immunity refers to the ability of innate immune cells to mount an enhanced subsequent response to diverse microbes, a phenomenon whose underlying mechanisms are under intense investigation.

IL-17 is associated with a protective role against infection with clinically virulent Mtb isolates (Gopal et al., 2014) and enhanced protection in mouse models (Aguilo et al., 2016). However, BCG delivered systemically is not a strong inducer of Th17, one potential explanation being that BCG strains lack the region of difference 1 (RD1) region (Dockrell and Smith, 2017), resulting in loss of the protein secretion system ESAT-6 that governs phagosomal rupture and host cell lysis. In fact, when complemented with the ESAT-6 containing RD1 region, BCG shows improved protective efficacy and enhanced Th17 responses in mice (Chatterjee et al., 2011). More recently, local pulmonary BCG administration via endobroncheal instillation in a rhesus macaque Mtb challenge model induced mucosal protective immunity mediated by Th17 polyfunctional cells and IgA production (Dijkman et al., 2019).

In contrast to cell-mediated immunity, the human humoral response against Mtb has been conventionally thought to exert little immune control over the course of Mtb infection or in response to BCG vaccination (Jacobs et al., 2016), due to the paradigm that humoral immunity plays little role in the protection against intracellular pathogens. However, the contribution of BCG vaccination specific Abs to specific and non-specific protection is a revived area of interest (see Box 1).

**Box 1. Vaccine-induced antibody-mediated immunity against mycobacteria.**

The ‘central dogma’ of anti-mycobacterial immunity outlines that T cell production of IFNγ activates macrophages to kill intracellular Mtb. Accordingly, measurement of IFNγ produced by T cells is the most widely used method for detecting immune responses following infection or vaccination with BCG (Nunes-Alves et al., 2014). Current strategies to develop next generation BCG vaccines are generally focused on the enhancement of IFNγ production by CD4+ T cells (i.e., Th1 cell-mediated immunity) (Achkar and Casadevall, 2013). Recent attention has been focused toward understanding the role, if any, of vaccine-induced antibodies (Abs) to prevent infection (Izzo, 2017). Various reported mechanisms of Ab-mediated protection against Mtb include direct antimycobacterial activity, opsonization, activation of complement, clearance of immunomodulatory mycobacterial antigens, increase of macrophage Ca2+ signaling, release of oxidants enhancing intracellular killing and other mechanisms of enhancing cell-mediated immunity (Achkar et al., 2014). How and whether BCG vaccination specific Abs may contribute to protective mechanisms remains unclear (Lu et al., 2016). Indeed, maternal infection with Mtb, and subsequently maternal Abs, do not seem to play a role in protecting neonates and young infants against mycobacterial infection, although maternal Abs inhibited purified protein derivative (PPD)-specific T cell responses in BCG vaccinated infants (Mawa et al., 2015). However, recent studies in mice (Ai et al., 2013; Alvarez et al., 2013) and humans (Zimmermann et al., 2016) have indicated a potential role for IgA Abs. Currently, the most compelling evidence for human IgG Ab-mediated immunity against mycobacteria may come from studies investigating IFN-independent markers of mycobacterial exposure. When compared to subjects with classic latent Mtb infection, Mtb ‘resisters’ display enhanced Ab avidity and distinct Mtb-specific IgM and IgG Fc profiles (Lu et al., 2019). BCG may also enhance Ab responses and, in some cases, T cell responses to other early life vaccines, such as hepatitis B, pertussis, and pneumococcal vaccines (Ota et al., 2002; Ritz et al., 2013; Scheid et al., 2018). Overall, understanding formulation-specific BCG-induced responses may necessitate complimentary investigation of functional Ab responses to vaccination, which may prove to be as important as inducing T cell production of IFNγ and/or heterologous responses. Such studies will shed fresh light on the mechanisms of BCG-induced protection and may inform development of next generation TB vaccines.

### Route of BCG Administration

The route of BCG administration can affect immune responses. Intradermal injection is the most common method of BCG vaccination and the route currently recommended by the WHO. Percutaneous administration is the only route licensed for use of BCG (Tice strain) as a TB vaccine in the United States. Given the more unpredictable nature of percutaneous administration, percutaneously administered formulations are manufactured to contain more colony forming units (CFU) compared to those meant for intradermal administration. A human adult randomized trial comparing the two methods showed that percutaneous BCG Tice vaccination was associated with lower reactogenicity, immunogenicity (as measured by lymphoproliferative responses) and delayed hypersensitivity responses (assessed using the mean size of PPD response) compared to intradermal vaccination (Kemp et al., 1996). Of note, the CFU dose for intradermal use of BCG Tice was adjusted in this study by diluting BCG, to match the WHO’s standard recommended dose. A later study comparing intradermal vs. percutaneous BCG Japan administration found significantly greater Thl cytokine and lymphoproliferative responses with percutaneous BCG (Davids et al., 2006). This study involved an infant cohort and CFUs were not adjusted for route of administration. Divergent results across studies may be related to the strain used (Tice vs. Japan) and also raise concerns about the administration routes of the vaccine, both of which have their challenges: percutaneous administration results in variable delivery of CFU subject to skin penetration, while intradermal delivery requires training and skill for optimal execution. Interestingly, a randomized trial in South African infants vaccinated at birth with intradermal vs. percutaneous BCG Japan found an equivalent incidence of TB over 2 years, questioning the relevance of administration route to clinical efficacy, though heterologous effects were not specifically assessed in this study (Hawkridge et al., 2008). A recent study in non-human primates demonstrated that intravenous administration of BCG provided 90% protection against TB as compared to the conventional intradermal route. Further studies of route of BCG administration are needed to inform optimal administration to humans (Darrah et al., 2020).

## Overview of Different BCG Vaccine Strains

### The Evolution of BCG Strains

**FIGURE 1:**
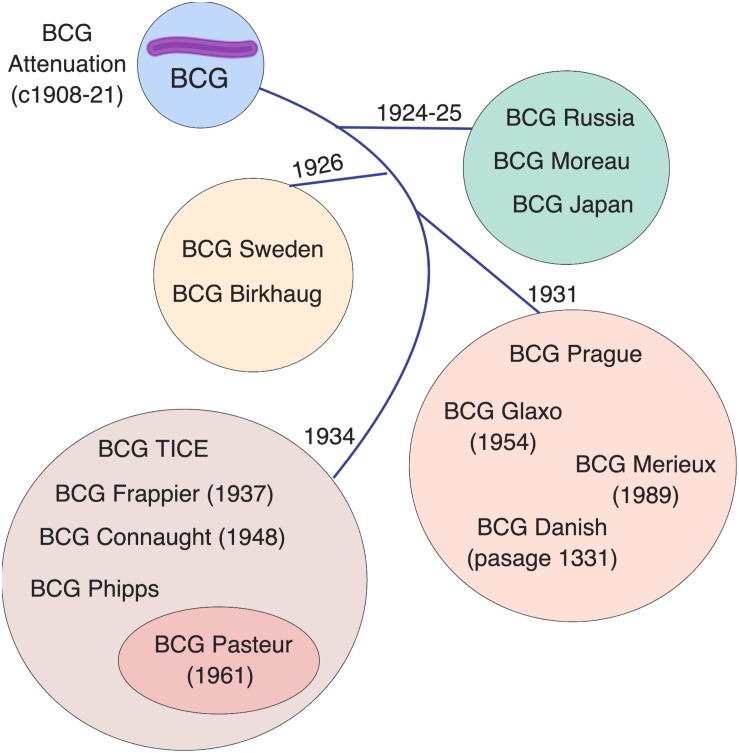
Licensed BCG formulations are derived from a parent strain developed in Paris, France. Multiple sub-strains have been generated using diverse culture methods, classified by genomic sequencing, resulting in a genealogy/timeline of BCG vaccine strains. Such BCG sub-strains differ in colony morphology, growth characteristics, biochemistry, immunogenicity, and virulence. The French (Pasteur) strain 1173 P2, Denmark (Statens Serum Institute) strain 1331, Glaxo strain 1077, Japan/Tokyo strain 172-1, Russian strain BCG-I, and Moreau RDJ, account for >90% of the BCG vaccines in use worldwide. The scheme depicts the distribution of vaccine formulations into four main groups (circles) based on their tandem duplication 2 (DU2) variant, which distinguishes the early (DU group I) from the late (DU group II-IV) vaccines. The lines indicate the chronology of derivation for each group. Modified from Brosch et al. (2007).

BCG is not a single vaccine, but rather a family of historically evolving and divergent vaccine formulations, further complicating the crucial task of defining mechanisms of action for these vaccines and their correlates of protection. The basis of BCG attenuation was the deletion of the genomic region RD1, which is absent from all *M. bovis* BCG strains, resulting in loss of the protein secretion system ESAT-6 that governs phagosomal rupture and host cell lysis (Mahairas et al., 1996; Brosch et al., 2007). Since its introduction in 1921 (Calmette, 1931), BCG seed lots were distributed globally for vaccine production at multiple sites. Based on historical records and phylogeny derived through molecular typing, a genealogy of BCG strains has been established, demonstrating the temporal relationship of their production and their dichotomy into “early” strains (e.g., Japan, Russia, Moreau, Sweden) and “late” strains (e.g., Pasteur, Tice, Denmark, Glaxo) (Behr and Small, 1999; Brosch et al., 2007; Abdallah et al., 2015). Before freeze-dried seed lots were derived from a single spreading colony in the 1960s, BCG strains were sub-cultured in different laboratories, yielding minority subpopulations that can impact virulence (Kroger et al., 1994), immunogenicity (Davids et al., 2006; Aguirre-Blanco et al., 2007), viability (Gheorghiu and Lagrange, 1983), colony size/counts and heterologous effects (Shann, 2015). BCG has continued to change with *in vitro* passage, resulting in further genetic diversity among strains (Figure 1). Comparative genome and transcriptome analysis of representative early and late BCG daughter strains, such as BCG Japan and BCG Pasteur respectively, has shown amplification of polymorphisms such as the tandem duplication DU2 in the later strains with implications for the expression level of known surface proteins and immunodominant prominent antigens (Brosch et al., 2007). The potential influence of these differences on the protective efficacy, immunogenicity, safety and heterologous effects of BCG immunization has generated considerable challenges for international TB immunization initiatives and highlights the importance of future studies comparing the different licensed BCG formulations (Wu et al., 2007; Ritz et al., 2008, 2012; Hayashi et al., 2009; Biering-Sorensen et al., 2015; Shann, 2015).

### Challenges in BCG Propagation *in vitro*

BCG strains supplied for clinical use vary depending on the original seed strain. Different culture or manufacturing conditions likely result in different genotypes within the same strain (Behr and Small, 1999) as well as epigenetic changes, even within a single genotype (Biering-Sorensen et al., 2015). Further variation may have occurred over time after a lab acquired the source and before freeze-drying, resulting in batch effects (Biering-Sorensen et al., 2015). Issues of batch or vaccine strain variability have proven very challenging to study at scale, as the EPI program has historically employed different vaccine strains as well as different batches of the same vaccine strain within the same region. Due to strain divergence and subsequent evolution, it has been difficult to assess the various bacterial strains using a single, consistent approach.

Over 14 different licensed BCG vaccine formulations comprised of distinct daughter strains of attenuated *M. bovis* are used globally with UNICEF being the largest supplier (UNICEF, 2015). Most countries import BCG from one of the international WHO prequalified manufacturers, while a few produce their own. However, there is no standardized culture methodology or one single culture medium recommended for the culture of BCG. This was demonstrated during the international collaborative study to evaluate and establish WHO reference reagents for BCG vaccine, where each of the 11 participating labs used their preferred culture medium to evaluate BCG candidate vaccines by culturable viable counts (e.g., Löwenstein-Jensen, Middlebrook 7H11 or 7H10, Ogawa, and Dubos) (Markey et al., 2009). Results between labs were highly variable, though reportedly within expected ranges, and may be partially attributable to challenges in standardizing colony counting due to variable colony sizes and the clump-forming nature of *M. bovis*.

Minor differences in production techniques can have profound effects on BCG growth (Shann, 2015). For example, inconsistent production methods may result in both type-by-type (e.g., BCG Denmark vs. BCG Russia) and lot-to-lot variability that can affect clinical efficacy. Growth and phenotypes of *M. bovis* BCG can be significantly influenced by the choice of media and the duration of culture incubation. For example, shorter time to detection of colonies was observed for *M. bovis* isolated from bovine tissues grown on 7H11 versus egg-based media (Corner et al., 2012). A study compared the immunogenicity of BCG vaccine grown in 7H9 medium, the most commonly used medium in laboratory studies, against that grown in Sauton medium, which is used for growing BCG by some manufacturers. This study showed clear differences in the efficacy of BCG grown in these different culture media, including variation in persistence within macrophages *in vitro*, apoptosis of infected cells, as well as cellular and humoral immune responses in mice *in vivo* (Venkataswamy et al., 2012). However, this study was largely limited to the BCG Pasteur strain, which might have behaved differently than other formulations, and did not examine specific BCG growth characteristics across culture media. Variable components between commercially available Oleic Albumin Dextrose Catalase (OADC) enrichment supplements can stimulate or inhibit the growth of mycobacteria and influence performance of Middlebrook 7H11 medium (Butler et al., 1990). Discrepancies in culture growth may alternatively indicate differences in viability after lyophilization or reconstitution. Slower growth has been associated with inocula that contain fewer viable bacilli (Corner et al., 2012). The number of live bacilli in the vaccine product decreases with time (Messina et al., 2018), as does survival after freeze-drying. Lastly, divergence in growth between BCG formulations may indicate unique nutrient needs, as BCG strains vary in their ability to catabolize amino acids, which act as the nitrogen source for BCG growth (Chen et al., 2003).

The presence and selection of minority populations within strains has been demonstrated by serial subculture under experimental conditions (Osborn, 1983) and is partially attributed to maintenance procedures of BCG lines. Specifically, BCG Tokyo 172 (the mother strain of BCG Japan, derived from the Pasteur strain in 1925), and Denmark 1331 (derived from the Pasteur strain in 1931), have a minority population of non-spreading colonies, as did BCG Pasteur before the seed lot system was introduced in 1961 (Osborn, 1983). Non-spreading colonies are characterized by opacity and lack of orientation. In contrast, in spreading colonies organisms have the tendency to adhere to one another in the direction of their long axis, and appear as a dense and opaque center surrounded by a halo, which consists of serpentine strands folded close together. BCG Japan substrains differ in cell wall lipid composition and antigenicity (Naka et al., 2011), with phenolic glycolipid and phthiocerol dimycocerosate found only in the substrains forming smooth colonies but not in those forming rough colonies. BCG Russia, the first documented daughter strain distributed by Institut Pasteur to Russia in 1924, is a natural *recA* mutant, preventing its genomic evolution (Keller et al., 2008) with unclear effects on BCG culture growth and the vaccine’s protective efficacy. BCG Russia is associated with lower effectiveness against tuberculosis, and lower frequency of BCG scars than BCG Denmark and BCG Japan. Of note, the genome of the BCG Russia strain features variably sized deletions of the polyketide synthase 12 (*pks12)* gene, necessary for β-phosphomycoketide production and the CD1c-mediated T cell response (Abdallah et al., 2015), potentially directly affecting immunogenicity of the daughter strains (Matsunaga et al., 2004).

### Different BCG Formulations Can Induce Distinct but Broad Ranges of Immunologic Responses in Humans

It is not currently known which BCG strain/formulation offers the best protection from TB disease, as immune correlates of protection are lacking. This limits inferences from *in vitro* studies. However, *in vitro* immunological and microbiological studies could provide critical insight in divergence of essential properties of the different strains, such as viability and host immune activating potential (Angelidou et al., 2020). Even in the absence of a defined correlate of protection these outcomes are probably critical to protection against TB.

Human data on cytokine induction after BCG administration are inconclusive as existing studies have low numbers of study participants, heterogeneous study designs, and variable formulations are tested with incomplete information on which formulation was used. As outlined in Table 1, *comparative studies were largely incomplete in terms of comparing all available strains or formulations.* Even though some patterns emerge such as BCG Denmark and BCG Japan perhaps being more immunogenic, generalizability of conclusions is difficult due to heterogeneous study designs, variable formulations, study populations, assays performed and endpoints studied. In a Mexican neonatal cohort vaccinated with BCG Denmark, Brazil (derived from BCG Moreau) or Japan, Mtb-specific recall immune responses after 1 year were examined (Wu et al., 2007). Upon activation of peripheral blood mononuclear cells (PBMCs) with Mtb proteins, BCG Denmark- or Brazil-immunized newborns demonstrated mRNA expression of cytokines important to adaptive immunity (IL-12, IL-27, IFNγ), while BCG Japan preferentially induced cytokines associated with acute inflammatory responses (IL-1α/β, IL-6, IL-24) (Wu et al., 2007). A randomized controlled trial in Australia showed that BCG Denmark and BCG Japan given at birth induced higher proportions of mycobacterial-specific polyfunctional [IFNγ(+)TNF(+)IL-2(+)] CD4 T cells than BCG Russia (Ritz et al., 2012). The impact of different BCG strains on the ontogeny of vaccine-specific and heterologous vaccine immunogenicity in the first 9 months of life was also examined in two African birth cohorts (Kiravu et al., 2019), where BCG Denmark vaccinated infants mounted significantly higher frequencies of polyfunctional CD4+ T cells, compared with infants vaccinated with BCG Bulgaria and BCG Russia. BCG-naïve adult volunteers immunized with BCG Denmark showed divergent whole blood pro-inflammatory and regulatory T cell responses, with significant induction of polyfunctional [IFNγ(+)TNF(+)IL-2(+)] CD4 T cells and IFNγ production confined to individuals with strong local skin inflammation, compared to regulatory-like CD8 T cell induction in individuals with mild skin inflammation (Boer et al., 2015). Polyfunctional CD4 cells have been associated with enhanced Th1 cytokine production and implicated as memory cells responsible for antigen-specific long-term protection (Darrah et al., 2007). However, whether their presence correlates with protective immunity remains highly controversial. In the limited studies done, there is no direct evidence that genetic variation of the vaccine strains accounts for the variability in efficacy and/or protection against TB based on the year a particular vaccine strain was given (Mangtani et al., 2014). Overall, these observations indicate that further clinical studies directly comparing different licensed BCG formulations/strains currently in use are needed to address these questions.

**TABLE 1:** Summary of human infant studies of BCG-induced innate, heterologous and mycobacteria-specific immunity.

Endpoints studied	BCG formulation comparisons	Geographic location	Assay	References
Recall responses, adaptive immune cytokines	Denmark > Japan	Mexico	PBMCs	Wu et al., 2007
Ab and cytokine responses to other vaccines	Pasteur > control	Gambia	PBMCs	Ota et al., 2002
Recall responses, IFNγ	Denmark > control	Gambia	PBMCs	Vekemans et al., 2004
Innate and recall cytokine responses	Pasteur	Papua New Guinea vs. Western Australia	PBMCs	van den Biggelaar et al., 2009
NO, IL-1β, IL-6, IL-8, IL-12, TNF in presence of IFNγ	Early strains (Russia, Moreau, Japan, Sweden, Birkhaug) > Late strains (Denmark, Glaxo, Mexico, Tice, Connaught, Montreal, Phipps, Australia, Pasteur)	Not applicable	Human epithelial cell-line A549, THP-1 cells	Hayashi et al., 2009
T cell frequency and cytokine profile	Japan at birth > control, Denmark for *in vitro* stim	South Africa	Whole blood	Kagina et al., 2010
Mycobacteria-specific and non-specific immune responses, scarification	Pasteur	Indonesia	Whole blood	Djuardi et al., 2010
TB-specific T cells, Th1 cytokines	Denmark = Japan > Russia	Australia (RCT)	Whole blood	Ritz et al., 2012
Mycobacteria-specific and non-specific immune responses, scarification	Denmark > Bulgaria > Russia	Uganda	Whole blood	Anderson et al., 2012
Maturation of innate responses to mycobacteria over first 9 months of life	Formulation not specified	South Africa	Whole blood and PBMCs	Shey et al., 2014
PPD responses, scarification	Denmark batches: slow growth > normal growth	Guinea Bissau (RCT)		Biering-Sorensen et al., 2015
T-cell immunity	Early Denmark (birth) = late Denmark (2 months)	Australia (RCT)	Whole blood	Ritz et al., 2016
Neonatal mortality	Russia = control	India (RCT)	–	Jayaraman et al., 2018
Mycobacteria-specific and non-specific immune responses	Denmark > Bulgaria = Russia	Nigeria, Cape Town	Whole blood	Kiravu et al., 2019
All cause hospital admissions, mortality, PPD responses, scarification	Denmark > Russia Japan > Russia	Guinea Bissau (RCT)	–	Schaltz-Buchholzer et al., 2019

## BCG-Induced Heterologous Effects

Human newborns are highly susceptible to infection due to functionally distinct innate (Kollmann et al., 2012) and adaptive immune responses (Kollmann et al., 2017) compared to other age groups. Epidemiologic studies have linked early life BCG immunization to an unanticipated reduction (∼50%) in all-cause mortality, which greatly exceeds a reduction in mortality attributable to TB (Higgins et al., 2014; Jensen et al., 2015). These observations suggest BCG induces heterologous protection against antigenically diverse, unrelated pathogens. One of the suggested mechanisms for heterologous protection against infection in the context of BCG vaccination is innate immune memory, also known as “trained immunity” (Netea et al., 2011).

### The Concept of Trained Immunity

**FIGURE 2:**
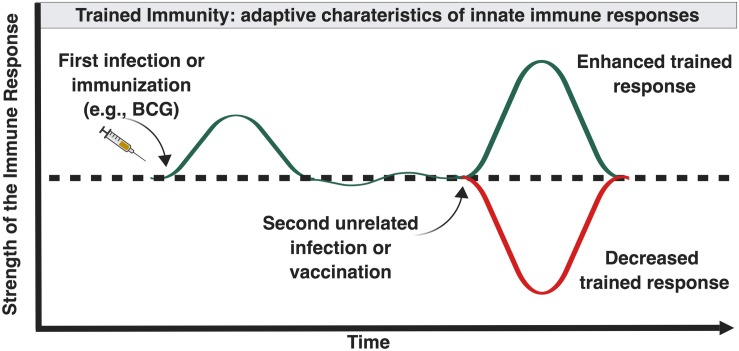
Influence of “trained” immunity on the magnitude of immune responses later in life. Certain forms and combinations of early life immune-stimulation, including BCG, can induce epigenetic changes in innate immune cells that can enhance or inhibit innate immune responses following future exposure to diverse antigenically unrelated pathogens (Netea and van der Meer, 2017).

Trained immunity is the ability of innate immune cells to mount an altered response against infection following a previous unrelated infection or vaccination (Figure 2). Innate immune memory is well described in plant immunology and invertebrates which lack adaptive immune response mechanisms (Kurtz, 2005). In contrast to adaptive memory mediated by B- and T-cells, innate memory primarily involves mononuclear phagocytes. Mammalian studies suggest that the innate host defense of vertebrates possesses similar properties. Vaccination of mice with BCG protects against secondary infections with *Candida albicans* or *Schistosoma mansoni* through activation of tissue macrophages (van ’t Wout et al., 1992). Injection of attenuated strains of *Candida* in athymic mice induced protection toward virulent *Candida* strains but also toward *Staphylococcus aureus*, through macrophage activation and proinflammatory cytokine production (Bistoni et al., 1986). Human innate immunity also exhibits immunological memory mediated by epigenetic and metabolic reprogramming of innate immune cells and their bone marrow precursors (Netea et al., 2019).

### Examples of Trained Immunity in Human Cohorts and Mechanistic Insights

Several observations in human epidemiologic studies support the notion that trained immunity occurs in the human neonate. One observation that supports a role for trained immunity in early life is the association of bloodstream infections in critically ill preterm newborns with enhanced pathogen-specific mononuclear cell PRR expression in the setting of subsequent Gram-positive or Gram-negative bacteremia (Zhang et al., 2010). This finding suggests that the neonatal innate immune system can remember previous activation such that responses to subsequent microbial challenges are altered. Similarly, histologic chorioamnionitis affecting preterm infants is associated with a significantly reduced risk of late onset sepsis, both with coagulase-negative Staphylococcus (most common) and other bacteria (Strunk et al., 2012), implying that perinatal inflammation may enhance functional maturation of the preterm immune system.

Immunization of human newborns may also trigger trained immunity. In observational studies in Guinea-Bissau, BCG vaccine had beneficial effects on overall mortality compared to no/delayed BCG vaccination (Kristensen et al., 2000), especially during the first 2 months of life (unadjusted MRR 0.74, adjusted MRR 0.55). Near halving of neonatal mortality in low-birth weight children vaccinated with BCG at birth was replicated in two subsequent randomized-controlled trials (Aaby et al., 2011; Biering-Sorensen et al., 2012). The reduction in neonatal mortality was associated with fewer cases of neonatal sepsis, respiratory infections and fever (Aaby et al., 2011). In another randomized-controlled trial between 2008 and 2013 including 2,320 low birth weight children, BCG given early (at birth) vs. late (>2.5 kg or when infant was 2 months old per the established practice) conferred a rapid survival benefit as early as 1 month of age (MRR 0.55), which was sustained up to 1 year of age (MRR 0.83) (Jensen et al., 2015). In the same trial, early BCG immunization led to increased production of Th1 polarizing and monocyte-derived pro-inflammatory cytokines, particularly IL-1β, IL-6, TNF and IFNγ, upon heterologous challenge of the infants’ whole blood *in vitro* with TLR-2, -4 or -7/8 agonists, or PPD, demonstrating a potentiating effect on innate cytokine responses (Jensen et al., 2015).

In addition to reduced mortality, heterologous beneficial BCG effects include decreases in infectious morbidities. Case control studies in Guinea-Bissau suggest that BCG vaccination and the presence of a scar among BCG-immunized infants was associated with a reduced risk of acute lower respiratory infection (ALRI) compared to unimmunized controls, with the association being stronger for females (Stensballe et al., 2005). In fact, children with ALRI were ∼3-fold more likely to have not received BCG vaccine compared to children without ALRI. Similar results were found in an exploratory analysis of national health survey data from 33 low- and middle-income countries between 2000 and 2010, where 0–5 year-old BCG vaccinated children had 17–37% lower risk of suspected ALRI compared to unvaccinated controls (Hollm-Delgado et al., 2014). A retrospective epidemiologic study in Spain used data from the Official Spanish Registry of Hospitalizations to identify differences in hospitalization rates in BCG-vaccinated children (Basque Country, where universal neonatal BCG vaccination is practiced) as compared to non-BCG-vaccinated children (rest of Spain, where BCG is not routinely used) (de Castro et al., 2015). Analysis of 464,611 hospitalization episodes over a 15-year period showed that neonatal BCG immunization was associated with fewer hospitalizations for respiratory infections (the preventive fraction, defined as the attributable proportion of disease cases prevented by BCG exposure, was 40% and statistically significant among all age groups) and sepsis (preventive fraction 36%, statistically significant among the infant group) (de Castro et al., 2015). Differences diluted with age suggesting a time-limited protective effect of BCG vaccination vs. lower rates of hospitalization for respiratory infection in older children. No significant differences in the already low mortality rates were observed.

BCG scarring has been correlated with heterologous protective effects. A recent prospective study in rural Guinea-Bissau showed that children vaccinated with the BCG Moscow strain (also known as BCG Russia) who developed a scar had 26% lower mortality compared to children who did not develop a scar, mainly attributable to prevention of deaths from respiratory infections (mortality rate ratio [MRR] 0.2) (Storgaard et al., 2015). This correlation of BCG scarring and improved survival has been replicated over different time periods and with different BCG strains; however, scarification rates differ by BCG formulation. For example, BCG Russia is less likely to produce a scar compared to BCG Japan and Denmark (Frankel et al., 2016; Funch et al., 2018). BCG-induced scarring in Ugandan newborns was associated with higher IFNγ responses to heterologous stimuli (tetanus toxoid, phytohaemagglutinin) at 1 year, and differed across strains (93% with BCG Denmark vs. 64% with BCG Bulgaria vs. 52% with BCG Russia) (Anderson et al., 2012). An RCT in Guinea-Bissau showed increased scarring induced by BCG Denmark and Japan compared to BCG Russia, but no significant differences in morbidity and mortality, at least by 6 weeks of age (Schaltz-Buchholzer et al., 2019), possibly because BCG Russia also induced relatively high scarification rates in this cohort compared to others. Even though development of scarring also depends on additional factors such as vaccination technique, preservation of the cold chain, nutritional status of the recipient, age at time of vaccination and prior exposure to non-tuberculous mycobacteria, variable scarification rates may still predict variable heterologous protection in populations vaccinated with different BCG formulations.

In adults, BCG immunization induces specific epigenetic markers associated with the acquisition of a trained or tolerant phenotype after BCG vaccination (Saeed et al., 2014). In healthy volunteers BCG induces trained immunity and heterologous protection from infections through epigenetic reprogramming of monocytes (Kleinnijenhuis et al., 2012), specifically trimethylation of histone H3 at lysine 4 (H3K4me3) at the level of cytokine and TLR4 promoters. To further characterize BCG-induced innate immune regulation, adult PBMCs were cultured with BCG *in vitro*. Following heterologous stimulation with TLR ligands and bacteria, there was increased production of TNF, an effect mediated through the Nucleotide-Binding Oligomerization Domain Containing 2 pathway (Kleinnijenhuis et al., 2012). In a randomized placebo-controlled adult study, yellow fever virus vaccine recipients who had been BCG vaccinated with the Denmark strain 1 month prior, had significantly lower yellow fever viremia compared to subjects who had received placebo vaccination (Arts et al., 2018). BCG vaccination conferred protection against yellow fever experimental infection by inducing genome-wide epigenetic reprogramming of monocytes involving genes related to signal transduction molecules, epidermal growth factor receptor, fibroblast growth factor, and vascular endothelial growth factor signaling pathways, as well as genes such as AKT1, MAPKs, and PI3K-related that have been shown to be important in β-glucan-induced trained immunity, the prototypical trained immunity-inducing agonist *in vitro*. This effect correlated with induction of cytokine responses indicative of trained immunity: higher pro-inflammatory cytokine production (TNF, IL-1β, IL-6) from BCG-vaccinated volunteers, compared to placebo-treated individuals, with a crucial role for IL-1β production and release. These observations suggest potential mechanisms for heterologous protection that could also apply to infants, as epidemiological studies have shown that BCG vaccination results in lower all-cause mortality in infants (Roth et al., 2006).

More recently, immune-gene priming long non-coding RNAs (lncRNAs), positioned at the nexus of RNA, DNA, and protein interactions, have emerged as key regulators of gene transcription in trained immunity by positioning themselves at the nexus of RNA, DNA, and protein interactions. Taking advantage of the three-dimensional nuclear architecture and the close proximity of functionally related immune genes in topologically associated domains (TADs), lncRNAs contribute to accumulation of H3K4me3 at the promoters of trained immune genes in human monocytes (Fanucchi et al., 2019).

Growing evidence that innate immune engagement by BCG enhances responses to other pathogens raises the possibility that some, or conceivably even most, of its clinical benefit is due to heterologous effects. However, the extent, mechanism and ontogeny of trained immunity in early life remain incompletely defined. Understanding how BCG-induced innate immune engagement, including BCG-induced enhancement of Th-polarizing cytokine production by antigen-presenting cells, varies by BCG strain and age is of basic and translational importance (Arts et al., 2015; Storgaard et al., 2015).

### The Role of Immunometabolism in BCG-Induced Trained Immunity

Intracellular metabolism plays key roles in regulating innate immune memory. In particular, different training programs induce metabolites that function as cofactors for epigenetic enzymes, which in turn induce chromatin and DNA modifications and modulate gene transcription upon re-challenge with a second stimulus (Netea et al., 2016). The *Warburg Effect*, first described in neoplastic cells, is a metabolic pathway important to trained immunity (Vander Heiden et al., 2009). Under normoxia, in resting cells, there is a low level of glycolysis and preferential pyruvate oxidation in the mitochondrion (oxidative phosphorylation), which confers slow but very efficient ATP production. In activated and proliferating cells, there is a metabolic switch from a state of oxidative phosphorylation to a state of glycolysis, crucial for the induction of the histone modifications and functional changes underlying BCG-induced trained immunity (Arts et al., 2016).

Epigenetic and metabolic reprogramming of hematopoietic progenitors may account for the long-term maintenance of trained immunity (Mitroulis et al., 2018). Trained immunity affects myeloid cells as well as precursor cells of the innate immune system in the bone marrow (Mitroulis et al., 2018). Administration of β-glucan in mice induced selective expansion of myeloid stem and progenitor cells accompanied by a global increase in energy metabolism in bone marrow progenitors, particularly enhancement of cholesterol biosynthesis and glycolysis. Cytokine analysis in the bone marrow extracellular fluid revealed elevated IL-1β levels, important in shaping immunometabolism within the bone marrow. In a randomized placebo-controlled human BCG immunization study with subsequent yellow fever vaccine challenge, reduction of viremia was highly correlated with the upregulation of IL-1β, a cytokine associated with the induction of trained immunity, but not with the specific IFNγ response (Arts et al., 2018), supporting a key role for IL-1β as a mediator of trained immunity responses (Moorlag et al., 2018). In mice, access of BCG to the bone marrow reshaped the transcriptional landscape of hematopoietic stem cells resulting in preferential myelopoiesis vs. lymphopoiesis and generation of macrophages that provided improved protection against TB (Kaufmann et al., 2018).

Changes in glucose, glutamine and cholesterol metabolism enable maintenance and longevity of trained immunity via accumulation of immunologically active intermediate metabolites (Fok et al., 2018). Examples include: (a) cholesterol, which participates in cell membrane remodeling and increased sensitivity to subsequent stimuli, (b) succinate and fumarate, which antagonize histone demethylation and suppress anti-inflammatory genes, (c) acetyl-CoA, an essential substrate for acetylating processes, and (d) NAD^+^ which is important for epigenetic changes resulting in a switch from glucose to fatty acid oxidation during LPS-induced tolerance and sepsis-induced immune paralysis (Conti et al., 2019). Immunometabolic changes may be different between newborns and adults, reflecting the differential nutritional and metabolic needs of the two groups, as well as their distinct immune response to pathogens (Kan et al., 2018; Dreschers et al., 2019). Indeed the ontogeny of immunometabolism is an emerging and promising area of research (Conti et al., 2019) (see Box 2).

**Box 2. Applying systems biology to systems vaccinology.**

Systems vaccinology, the application of global molecular techniques such as metabolomics, proteomics, or transcriptomics, can provide unique insights into vaccine-induced immune responses by identifying molecular signatures that may predict and give insight into vaccine-induced immunogenicity and protection (Pulendran, 2014). The systems biology can provide valuable insights into host–pathogen interaction with Mtb as well as generate tools for early and proper diagnosis of TB, identification of BCG protective efficacy, and accelerated development of better TB vaccines. The metabolome, the inventory of all metabolites present in a given sample, reflecting both genetic and epigenetic influences, shifts upon immune activation and can in turn shape immune responses (O’Neill et al., 2016). Metabolic phenotype influences vaccine immunogenicity and together with orthogonal datasets can identify correlates of vaccine immunity (Li et al., 2017). Lipid metabolism is pivotal in the regulation of inflammatory signaling hence making lipidomics, an in-depth profiling of lipid metabolites, a valuable modality as well. Lipid metabolism regulates immune cells via cell membrane synthesis (Lochner et al., 2015) and is important to epigenetic reprogramming of immune cells (Kleinnijenhuis et al., 2014, 2015). Mass-spectrometry-based metabolomics, together with computational tools, can identify and correlate metabolic pathways between samples, providing a powerful approach for clinical diagnostics (Johnson et al., 2016). More studies are warranted to build the area of biomarker identification while addressing the challenges of identifying correlates of protection against TB.

## BCG-Mediated Immune Modulation of Autoimmune and Inflammatory Diseases

BCG has been recognized as a potent immunomodulator for decades with extensive use for cancer and particularly bladder cancer treatment (Rosenthal, 1988; Ravaud et al., 1990). In the past decade, there has been revived interest in BCG vaccine for potential new therapeutic uses in type 1 diabetes mellitus and treatment of other forms of autoimmunity. When administered to young NOD (autoimmune-prone) mice, BCG could not only stop new-onset diabetes but also reverse end-stage diabetes, owing to induction of suppressive regulatory T cell (Treg) expansion (Ryu et al., 2001; Kodama et al., 2003), thereby preventing the immune system from attacking the body’s own tissue. In a clinical trial involving humans with longstanding type 1 diabetes mellitus, repeat BCG administration (2 doses) led to transient restoration of pancreatic cell islet function *in vivo* (for 4–6 weeks after vaccination) (Faustman et al., 2012). The suspected mechanism was BCG-induced proliferation of Tregs and selective elimination/suppression of auto-reactive cytotoxic T cells, possibly via TNF induction/TNF receptor 2 agonism (Faustman, 2018). Long-lasting improvements in glycemic control as evidenced by sustained decreases in hemoglobin A1c were achieved via accelerated glucose utilization induced by a systemic shift from oxidative phosphorylation to aerobic glycolysis (Kuhtreiber et al., 2018).

In a double-blind, placebo-controlled trial conducted in Italy involving subjects with early symptoms consistent with multiple sclerosis (MS), participants were randomly assigned to receive BCG or placebo and monitored monthly with brain Magnetic Resonance Imaging (MRI) (6 scans) (Ristori et al., 2014). By the end of the study, 58% of those vaccinated had not developed MS, compared with 30% of those who received placebo (Ristori et al., 2014). Overall clinical benefits after BCG administration in new onset MS were durable and even enhanced at 5 years. In another trial, BCG vaccination was found to decrease MS disease activity and prevent progression of brain lesions in patients with relapsing-remitting MS (Paolillo et al., 2003). A phase III clinical trial of BCG to reverse progression of MS is now underway.

BCG vaccination has also been associated with a reduced risk of atopic disorders as noted in a Japanese cohort (Shirakawa et al., 1997), as well as in African children, where the reduction in atopy associated with BCG was greater the earlier the age at vaccination, with the largest reduction seen in children vaccinated in the first week of life (Aaby et al., 2000). This observation is consistent with BCG being a powerful inducer of a Th1 phenotype in infants (Marchant et al., 1999) and shifting their immune response away from the Th2-type that is typically favored in early life. Importantly, these immune polarizing effects of BCG may be yet another result of trained immunity, which may contribute to host survival in early life and affect the risks of infection, allergic and chronic inflammation later in life (Levy and Netea, 2014). A randomized controlled trial to determine if BCG immunization at birth reduces allergy and infection in infants is currently underway in Australia (Melbourne Infant Study, NCT01906853).

## The Role of Immune Ontogeny in Shaping BCG-Induced Tb-Specific and Heterologous Immunity

Few studies have investigated the influence of age at and timing of immunization on BCG-induced immunogenicity and protection against TB. BCG-specific effector CD4 T cell responses demonstrate increased antigen-specific CD4 T cell proliferative capacity in infants compared to older children (Whittaker et al., 2018b). Vaccination at birth induces a broad Th1/Th2/IL-17/Treg anti-mycobacterial response but the Th1/Th17 response is reduced when delaying the vaccine from birth to 4 1/2 months of age (Burl et al., 2010). In a randomized trial of low birth weight newborns, BCG significantly increased *in vitro* whole blood cytokine responses to heterologous TLR agonists and to PPD in infants 4 weeks post-vaccination, particularly cytokines IL1β, IL-6, TNF, and IFNγ (Jensen et al., 2015), potentially contributing to broad protection against infections. These studies illustrate that timing of BCG administration can be crucial for its immunogenicity with distinct effects depending on which outcomes are studied (mycobacterial-specific vs. heterologous). Mechanistic studies are needed to provide a basis for understanding the impact of immune ontogeny on BCG immunogenicity.

Comparable CD4 and CD8 T cell anti-mycobacterial responses and whole blood cytokine production were noted in Australian infants who received BCG Denmark at birth (early BCG) compared to 2 months after birth (late BCG) (Ritz et al., 2016). However, in TB-endemic regions such as Cape Town and South Africa, delaying immunization with BCG Denmark 10 weeks post-birth led to increased frequencies of memory CD4 T cells at 1 year of age (Kagina et al., 2009). These two seemingly contradictory studies emphasize the importance of immune ontogeny, as well as genetic and epigenetic host factors, including prior and ongoing host exposure to non-tuberculous mycobacteria, to the immunogenicity of live vaccines (Plotkin, 2013).

BCG vaccination in children (Jensen et al., 2015) results in different cytokine induction patterns compared to adults (Kleinnijenhuis et al., 2012). Vaccine efficacy rates were indeed higher in studies conducted in populations vaccinated during childhood compared with populations vaccinated at older ages (Colditz et al., 1995). The longevity of BCG clinical effects remains largely unknown and may in part depend on age of immunization. In the largest community-based controlled trial of BCG vaccination conducted in southern India in the 1960s, vaccine recipients were reevaluated 15 years after BCG vaccination (Tuberculosis Research Centre, 2013): protective efficacy in persons who had been vaccinated as children was found to be 17%, while no protective effect was seen in people who had been vaccinated as adolescents or adults (Tuberculosis Research Centre, 2013).

WHO currently recommends BCG at birth for countries where TB is endemic since birth is the first point of contact for the newborn with the healthcare system. In practice, however, many healthcare systems continue to institute policies such that BCG is not administered unless a certain number of infants are present to receive immunization from the multi-dose BCG vial resulting in missed opportunities to administer it at the earliest possible age per WHO recommendations (Schaltz-Buchholzer et al., 2017). However, based on the above, a “one size fits all” policy on optimal BCG timing may not be realistic and immunization should be tailored to different global populations with different risk factors in different settings. Further investigations involving the ontogenetic aspects of BCG-induced immunogenicity and protection against TB are needed. Highly standardized comparison studies should account for the environmental (local and regional) exposure, genetic and epigenetic factors, biological age, and immunological status of vaccinated participants. Such studies would further inform the variation of heterologous effects seen as a result of BCG vaccination.

## BCG as a Model to Build Next Generation Vaccines

The ability of live vaccines such as BCG to induce heterologous immunity raises the possibility of leveraging such broadly protective effects in the development of novel vaccine formulations (Whittaker et al., 2018a), in the form of “trained immunity-based” vaccines (Sanchez-Ramon et al., 2018). Firstly, increased awareness of innate memory may be employed to define new classes of vaccine adjuvants (Topfer et al., 2015), crucial tools to optimize current vaccines and develop new ones (Dowling and Levy, 2015). Adjuvants enhance responses to vaccine antigens by a variety of mechanisms (Coffman et al., 2010), but like BCG, many are capable of acting via PRR signaling (e.g., TLRA), which possibly could hold the potential of inducing innate memory and could thereby mediate long-term changes in host defense. Also, recent advances in adjuvant discovery and delivery have opened up a new toolbox on how vaccinologists can employ adjuvants, including synthetic small molecule PRR agonists (Dowling and Levy, 2015). Thus, to confer protective immunity a strategy might be the combination of adjuvants, with potential of inducing beneficial non-specific trained immunity responses, formulated along with the specific selected antigen epitopes. An important aspect to take into account is that it is not yet known whether or not all PRR stimuli produce trained immunity-like responses. As different adjuvants may trigger different cell activation pathways and have age-specific activity, it is likely that more than one trained immunity pathway could be targeted for perturbation. In addition, putative target cell populations for innate training may vary, including progenitor cells, tissue resident or circulating monocytes, which may be optimally targeted via specific routes of administration or by rationally selected adjuvant formulations (Dowling and Levy, 2015; Nanishi et al., 2020).

Secondly, characterizing mechanisms by which BCG enhances neonatal immunity may inform rational design of scalable, synthetic subunit vaccine formulations for newborns. Initially, TLR7/8a imidazoquinolines were shown to induce trained immunity in newborn mice (Wynn et al., 2008), raising the possibility that such an approach could generate a vaccine that may also induce “BCG-like” trained immunity. However, since free un-formulated molecules may have off target effects, another approach is to build “BCG-like” synthetic “non-live” particulate vaccines that may mimic BCG’s immune-enhancing effects. Inclusion of an imidazoquinoline small molecule TLR8 agonist in a polymersome nanoparticle (∼150 nm diameter) induced robust Th1 polarizing responses from human newborn monocyte-derived dendritic cells *in vitro* that at least matched and for some biomarkers such as IL-12p70 exceeded those induced by BCG Denmark (Dowling et al., 2017). Of note, when co-loaded with the *M. tuberculosis* antigen 85B peptide 25, the TLR8-agonist containing polymersome nanoparticles were comparable to BCG in inducing antigen-specific T cell responses in human TLR8-expressing neonatal mice *in vivo* (Dowling et al., 2017). This is promising, since BCG reduces the risk of disseminated early life TB safely, elicits Th1-type neonatal immune responses and requires only a single dose at or shortly after the time of birth. The key role of TLR8 agonists for protection against Mtb challenge was recently verified by others with humanized TLR8 mice (Tang et al., 2017) and in human studies, wherein humans with hypermorphic alleles of TLR8 demonstrated enhanced BCG-induced protection against TB (Ugolini et al., 2018).

Thirdly, the robust safety and immunogenicity profile of BCG has rendered it an attractive vector for vaccine development against other infectious diseases (Hernandez-Pando et al., 2007; Bastos et al., 2009; Nieuwenhuizen and Kaufmann, 2018). Recombinant BCG technology has been studied in the context of vaccination against HIV (Aldovini and Young, 1991), Lyme disease (Stover et al., 1993), malaria (Matsumoto et al., 1998), measles (Zhu et al., 1997), and HCV (Uno-Furuta et al., 2003). When administered in early life, BCG can act as an adjuvant enhancing antibody responses to recombinant hepatitis B surface antigen (rHBsAg) both in mice and in human infants (Ota et al., 2002; Zimmermann et al., 2019). In another approach, a recombinant strain of *M. bovis* BCG that secretes high levels of functional murine monocyte chemotactic protein 3 (BCG_MCP–__3_) attenuated vaccine virulence in immunodeficient mice, while maintaining protective efficacy against Mtb in mice by enhancing antigen-specific IFNγ T cell responses, as compared to a control BCG (Pasteur strain 1173P2) (Ryan et al., 2007). A recombinant BCG strain expressing listeriolysin O to enhance cytosolic entry of BCG antigens for MHC I presentation, named VPM1002, induced both CD4 and CD8 responses and demonstrated safety and immunogenicity in a phase 2 clinical study in South African newborns (Loxton et al., 2017). Overall, insights into BCG vaccine-induced heterologous and specific immunity may provide insights into the development of a broad spectrum of anti-infective vaccine formulations.

## Conclusion

Despite nearly a century of use, policies and practices around BCG immunization vary widely across the world. Much remains to be learned regarding the relative protective efficacy of different licensed BCG formulations and it is important to ensure that BCG vaccines selected for use in large-scale immunization schemes maintain the stability of their characteristics. Our growing understanding of the distinct neonatal immune response and of innate immune memory in early life will increasingly inform optimal immunization in this age group. Epidemiologic studies suggest that the benefit of BCG vaccination may vary by BCG formulation and age of administration with optimal timing in early life to maximize both specific and heterologous beneficial effects. Future studies should directly compare licensed BCG formulations, including their optimal timing of administration, and measure both heterologous and specific protection in high mortality populations. Characterizing activation of age-specific immune responses by BCG strains and defining potential correlates of BCG-induced protection via correlation with known relative heterologous clinical benefit, can inform optimization of BCG’s use. This may involve potential BCG (re)introduction in national immunization schedules, BCG utilization in prime-boost schedules, use of BCG as a vector for other vaccinal antigens, as well as design of new vaccines that mimic BCG to harness innate immune memory for clinical benefit (Dowling et al., 2017).

## Author Contributions

AA conceived the manuscript. AA, JD-A, DD, and MC wrote the manuscript. DD created the manuscript figures. KS, SvH, DD, RH, and OL revised it critically for important intellectual content. All authors read and approved the submitted version.

## Conflict of Interest

OL and DD are named inventors on several vaccine adjuvant formulation patent applications. The remaining authors declare that the research was conducted in the absence of any commercial or financial relationships that could be construed as a potential conflict of interest.

## Footnotes

1 http://www.bcgatlas.org

## Abbreviations

Ab: antibody
ALRI: acute lower respiratory infection
BCG: Bacille Calmette-Guérin
DTP: diphtheria-tetanus-pertussis
EPI: Expanded Program on Immunization
lncRNAs: long non-coding RNAs
MRI: Magnetic Resonance Imaging
MRR: mortality rate ratio
MS: multiple sclerosis
Mtb: *Mycobacterium tuberculosis*
OADC: Oleic Albumin Dextrose Catalase
PBMCs: peripheral blood mononuclear cells
PPD: purified protein derivative
PRR: pattern recognition receptor
RD1: region of difference 1
TAD: topologically associated domain
TB: tuberculosis
TLR: Toll-like receptor
Treg: regulatory T cell
WHO: World Health Organization.
